# Return to normal pre-COVID-19 life is delayed by inequitable vaccine allocation and SARS-CoV-2 variants

**DOI:** 10.1017/S0950268822000139

**Published:** 2022-01-24

**Authors:** Feng Liu, Zebin Zhao, Chunfeng Ma, Xiaowei Nie, Adan Wu, Xin Li

**Affiliations:** 1 Northwest Institute of Eco-Environment and Resources, Chinese Academy of Sciences, Lanzhou 730000, China; 2 University of Chinese Academy of Sciences, Beijing 100049, China; 3 State Key Laboratory of Tibetan Plateau Earth System, Resources and Environment (TPESRE), National Tibetan Plateau Data Center, Institute of Tibetan Plateau Research, Chinese Academy of Sciences, Beijing 100101, China; 4 The Alliance of International Science Organizations, Beijing 100101, China

**Keywords:** Administered vaccinations, COVID-19, delta variant, herd immunity, vaccine effectiveness

## Abstract

As a result of the COVID-19 pandemic, whether and when the world can reach herd immunity and return to normal life and a strategy for accelerating vaccination programmes constitute major concerns. We employed Metropolis–Hastings sampling and an epidemic model to design experiments based on the current vaccinations administered and a more equitable vaccine allocation scenario. The results show that most high-income countries can reach herd immunity in less than 1 year, whereas low-income countries should reach this state after more than 3 years. With a more equitable vaccine allocation strategy, global herd immunity can be reached in 2021. However, the spread of SARS-CoV-2 variants means that an additional 83 days will be needed to reach global herd immunity and that the number of cumulative cases will increase by 113.37% in 2021. With the more equitable vaccine allocation scenario, the number of cumulative cases will increase by only 5.70% without additional vaccine doses. As SARS-CoV-2 variants arise, herd immunity could be delayed to the point that a return to normal life is theoretically impossible in 2021. Nevertheless, a more equitable global vaccine allocation strategy, such as providing rapid vaccine assistance to low-income countries/regions, can improve the prevention of COVID-19 infection even though the virus could mutate.

## Introduction

Due to the persistence of the global outbreak of coronavirus disease 2019 (COVID-19), addressing the trade-off between containing the pandemic and returning to normal pre-COVID-19 life has become increasingly urgent [1, 2]. One solution to this dilemma is international cooperation with respect to vaccination [3]. As a result of the ongoing COVID-19 vaccination programmes, plans for returning to normal life are being implemented in a few countries. For example, the USA aimed to vaccinate 70% of adults by 4 July 2021 (achieved by 2 August [4]) with the goal of resuming social and economic activities similar to those of the pre-pandemic period [2]. The US economy is expected to show 6.4% growth in 2021, and this expectation conveys strong confidence in containing the pandemic and avoiding recession [5]. Vaccinated visitors were welcome to travel to the European Union in the summer of 2021, and unconditional free population movement among its member states was allowed [6]. However, due to the limited supply and inequitable allocation of vaccines, the rollouts of COVID-19 vaccines in most low-income countries are largely out of sync with those in high-income countries. By 12 September 2021, 76% of the 50 richest countries had administered more than 100 vaccine doses per 100 people, whereas 66% of the 50 poorest countries had not yet administered 10 doses per 100 people. This inequitable allocation of vaccines appears to be worsening because some countries with high vaccination rates have decided to offer COVID-19 booster vaccinations in the next few months [7].

The point at which humans can return to normal pre-COVID-19 life depends mainly on the emergence of global herd immunity. Although high vaccination rates can guarantee herd immunity, risks remain. Countries with high vaccination rates should be highly aware of the uncontrolled spread of the virus in other parts of the world. In addition, SARS-CoV-2 variants, i.e. the Delta variant, can reduce the effectiveness of the vaccines by 88% [8] to 66% [9]. As of 24 August 2021, cases of the Delta variant [10] had been reported in a total of 163 countries, particularly the USA, and the proportion of infections caused by the Delta variant was higher than 97% [11]. Whether virus variants might ultimately result in compromised herd immunity remains unclear. Thus, when and how the world will reach herd immunity and the potential barriers in the path to normal life are unknown.

In this study, to determine possible perspectives on the global COVID-19 pandemic, we conducted a modelling study using a series of scenarios based on the current vaccination strategy, a more equitable vaccine allocation strategy and different levels of vaccine effectiveness for SARS-CoV-2 variants. Our objective was to study whether the world can reach herd immunity and return to normal life in the coming years and, if so, at what cost. Furthermore, we discuss the implications of effective interventions to protect against this infectious disease.

## Methods

### Epidemic model

**Fig. 1.**
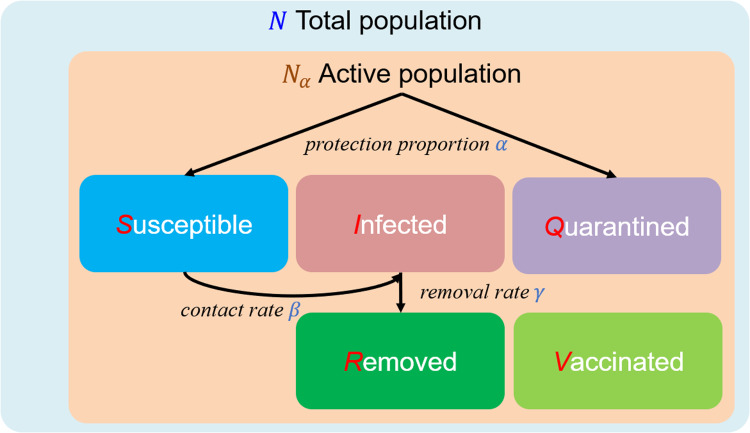
Five compartments of the SIRV model and their relationships with the parameters.

SIR-type models have been widely used for modelling the dynamics of the COVID-19 epidemic. We propose a susceptible-infected-removed-vaccinated (SIRV) model (Fig. 1) involving a two-dose vaccination strategy that is formulated as follows:1
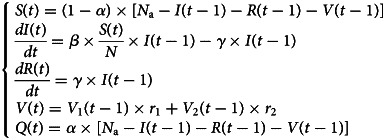
where *S*(*t*), *I*(*t*), *R*(*t*), *V*(*t*), *Q*(*t*), *N* and *N*_a_ represent the susceptible, infected, removed, vaccinated, quarantined, total and active populations, respectively. The active population *N*_a_ denotes the people who have the ability and are willing to contact others during all stages of the pandemic lifecycle. The parameter *α* denotes the protection proportion, and *β* and *γ* denote the rates of contact and removal, respectively. All parameters were estimated using an estimation method based on Metropolis–Hastings (M-H) sampling. *S*(*t*) and *Q*(*t*) are determined based on *α* in view of the control measurements. *V*_1_(*t*) and *V*_2_(*t*) are the numbers of people administered one dose and two doses, respectively, and *r*_1_ (ranging from 45% to 55% for SARS-CoV-2 and from 34% to 44% for the Delta variant) and *r*_2_ (ranging from 85% to 95% for SARS-CoV-2 and from 60% to 80% for the Delta variant) are the corresponding levels of vaccine effectiveness. The ranges of *r*_2_ are in accordance with the effectiveness of the vaccines against SARS-CoV-2 and the Delta variant [8, 9].

Although the vaccine expiration date is important for the quantitative prediction of the SARS-CoV-2 dynamics, the relevant data remain in the research phase [12]. Thus, this factor does not qualitatively affect our results, particularly in 2021. Long-lived immunity should not have an impact on the vaccine effectiveness included in Eq. (1).

### Herd immunity

Herd immunity refers to the sum of naturally acquired immunity and vaccinated immunity, which can govern the transition from COVID-19 to endemicity. In our study, the ratio of herd immunity to the total population is defined as *h* = [*I*(*t*) + *R*(*t*) + *V*(*t*)]/*N*, where *I*(*t*), *R*(*t*), *V*(*t*) and *N* are as in Eq. (1). The numbers of people who need to be vaccinated and to have recovered from COVID-19 to reach herd immunity are unknown. The exact value of *h* is currently unclear, and the model-based percentage of a population has been estimated to range from 60% to 90% [1] or can even equal approximately 43% through naturally acquired immunisation in an age-structured population [13]. The ratio equals 60% based on the basic reproduction number *R*_0_ of COVID-19, which equals 2.5 according to a report published in February 2020 by the World Health Organization-China Joint Mission on COVID-19 [14]. In this study, we used a higher herd immunity ratio of 70% to ensure a strong immunologic barrier.

### Parameter estimation

We used M-H sampling [15, 16] to trace the dynamics of the epidemic model shown in Eq. (1). Using the true infection data as the reference Ω, the posterior distribution of the unknown parameters *θ* conditioned on Ω is *P*(*θ*|Ω) ∝ *P*(Ω|*θ*)*P*(*θ*), where *P*(*θ*) is the prior parameters and *P*(Ω|*θ*) is the likelihood function. M-H sampling is a typical Markov chain-Monte Carlo (MCMC) algorithm used to calculate the intractable *P*(*θ*|Ω) parameter.

In our study, the sampling space comprises three parameters (protection proportion *α*, contact rate*β* and removal rate *γ*) and the active population *N*_a_. The corresponding sampling ranges in the SIRV model are *α* ∈ [0.01, 1], *β* ∈ [0.0001, 0.5], *γ* ∈ [0.000001, 0.1] and *N*_a_ ∈ [1, *N*]. Note that these figures should be suitable for modelling all countries/regions; thus, the exact ranges depend mainly on experience, and the algorithm might take more time to search for optimal parameters.

### Data source

This study was driven by epidemic data collected from the dashboard [17] of the Center for Systems Science and Engineering (CSSE) at Johns Hopkins University and the official website of Our World in Data [18]. The gross national income per capita data are based on the World Bank list of economies published in June 2020. We propose the timetable to reach herd immunity under the current phase of the pandemic and the numbers of vaccinations administered (biweekly averaged data from 30 August 2021 to 12 September 2021) and assume that the corresponding control measurements are invariant.

### Design of the experiments

**Fig. 2.**
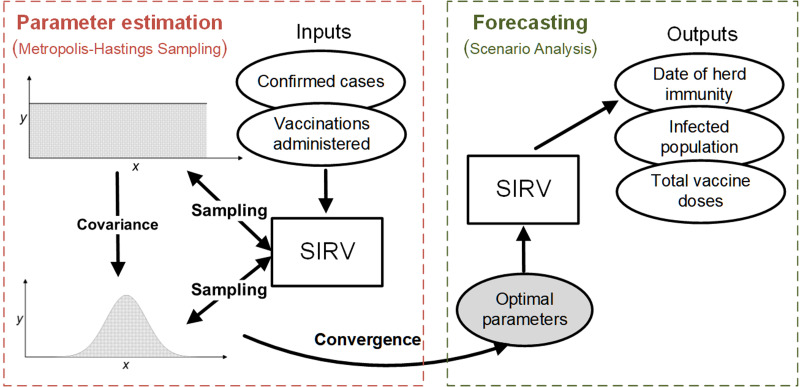
Flowchart of the implementation of epidemic forecasting based on the SIRV model and Metropolis–Hastings sampling. Parameter estimation is driven by the daily confirmed cases and the numbers of vaccinations administered at 2-week intervals. The optimal parameters are directly incorporated into the SIRV model to predict the dynamics of the infected and vaccinated populations.

The general framework of the real-world experiment for one country is presented in the flowchart in Figure 2. This flowchart consists of two parts. The first part involves parameter estimation based on M-H sampling. Here, the daily confirmed cases and current vaccinations administered are regarded as references for M-H sampling. A sample of parameter vectors according to uniform distributions in the sampling space is selected to determine whether it can be accepted according to the references. After sufficient samples are obtained, a covariance of the parameters is generated to formulate a normal proposal distribution in the sampling space. The optimal parameters are then collected within this normal distribution. In the forecasting part of the flowchart, the SIRV model is implemented using the optimal parameters, and the time of vaccination termination is defined at the time at which all members of the susceptible populations had received two doses of a vaccine.

As shown in Figure 2, the daily confirmed cases and current vaccinations administered serve as the inputs for the real-world experiment, and the outputs include three parts. The date of herd immunity is recorded if the herd immunity ratio is reached, i.e. the proportion of infected, recovered and vaccinated populations is higher than 70%. The total infected population is the sum of *I*(*t*) and *R*(*t*), and the total vaccine doses are equal to *V*_1_(*t*) + *V*_2_(*t*) × 2.

The same flowchart applies to the more equitable vaccine allocation scenario, and we use a loop to adjust the daily average rate of vaccination; i.e. if the date of herd immunity for one country is later than 31 December 2021, the vaccination strategy in the next loop adds an additional 1/30 of the current doses; otherwise, the same number of doses is subtracted. The loop terminates when the date of herd immunity is 31 December 2021.

### Model of the vaccine efficacy level

To achieve herd immunity goals, the total number of vaccine doses is estimated using Eq. (2):2
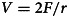
where *V* is the total number of vaccine doses, the number 2 indicates that the study involves a two-dose vaccination strategy, *F* is the fully vaccinated population, and *r* is the efficacy level of the vaccine. Equation 2 can be used to calculate the demands for vaccines with different efficacy levels.

### Cumulative case-to-vaccine dose ratio

This ratio is calculated as abs[(TV − CC)/TV], where TV denotes half of the total vaccine doses required, CC denotes the number of cumulative cases (Table S2), and abs is the absolute value function. A ratio  ≈1 indicates that herd immunity is reached mainly through vaccination; otherwise, infected people account for a significant proportion of the immune population. We use this ratio to identify countries with both severe pandemic situations and a limited vaccine supply.

## Results

### Scenario of current administered vaccinations

**Fig. 3.**
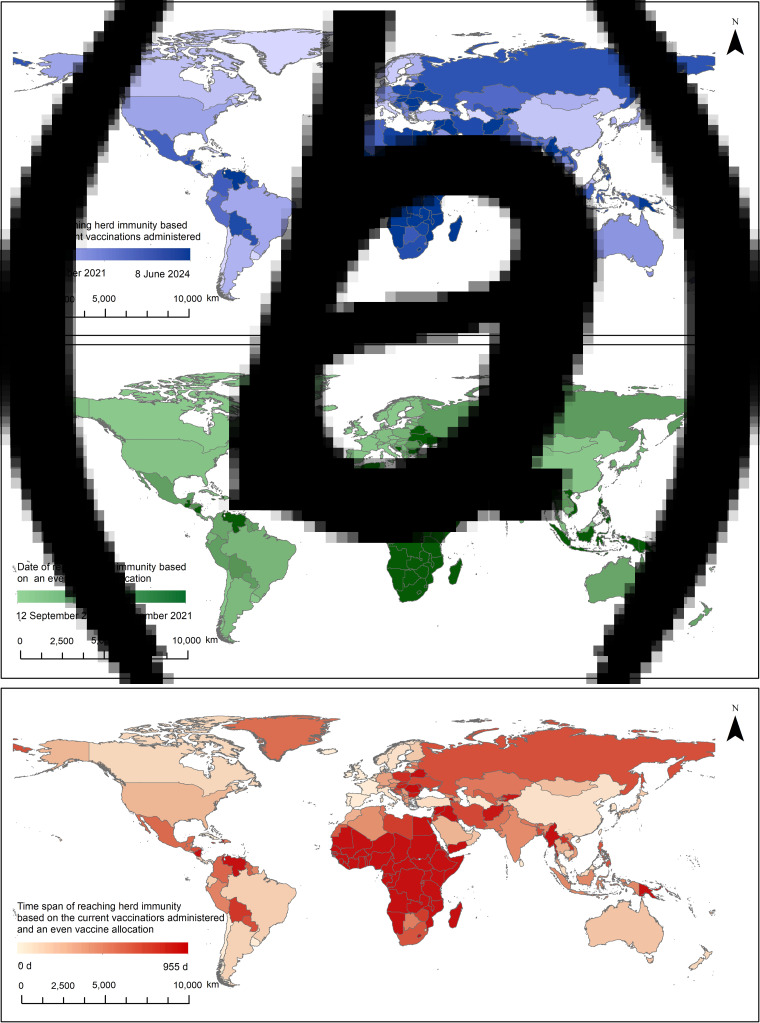
Timetable for herd immunity in 191 countries/regions. The timetable based on the current vaccinations administered (a) demonstrates that 58 countries/regions may face more than 3 years of vaccine shortages, and that based on a more equitable vaccine allocation strategy (b) demonstrates that all countries/regions could reach herd immunity by the end of 2021. Figure (c) shows the time span between (a) and (b).

**Fig. 4.**
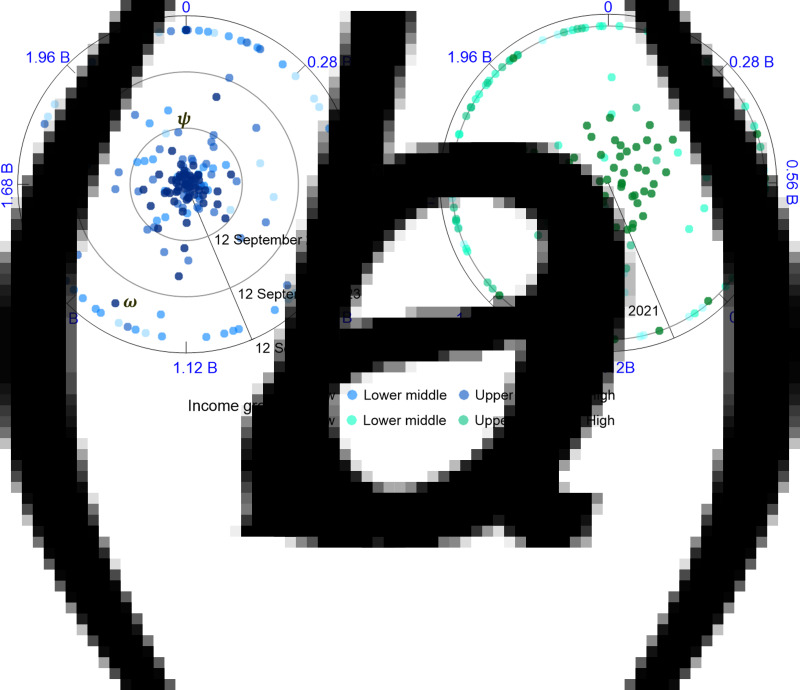
Polar plots based on (a) the current vaccinations administered and (b) a more equitable vaccine allocation strategy. The plots include the date of herd immunity (the radial axis) and the number of required doses of vaccines (the angular axis, billions). The dots represent countries that have four different levels of gross national income per capita. The poles of the plots correspond to the start date of 12 September 2021.

We first investigate the perspective of the global COVID-19 pandemic caused only by SARS-CoV-2, which means that the vaccine effectiveness against the virus after full vaccination is approximately 90%. This test follows the flowchart presented in Figure 2, and the timetable for reaching global herd immunity (Table S1) records the corresponding outputs for each country/region. According to the current vaccinations administered, the results show that only 61 countries will have reached herd immunity by the end of 2021 (Fig. 3a). In contrast, 58 countries – most of which are in Africa, South America, Eastern Europe and South and Southeast Asia – may face more than 3 years of vaccine shortages. The economic evidence shown in Figure 4a suggests more obvious heterogeneity among the countries/regions with different income groups. Most high-income countries can reach herd immunity in less than one year, but in low-income countries, the severe impacts of COVID-19 are likely to persist for years to come. The total required vaccine doses and the cumulative cases by the end of 2021 equal 8.90 billion and 261.01 million, respectively. Countries with larger infected populations, such as *ψ*in Figure 4a, usually need fewer vaccine doses than countries with smaller infected populations but experience more deaths. A few countries with high incomes but low vaccination rates, such as *ω* in Figure 4a, may largely show delayed global herd immunity because these countries are deeply engaged in global economic integration. This fragmented timetable suggests that global herd immunity may not occur for another 3 years.

However, in the absence of an adequate vaccine supply and of an appropriate allocation of vaccines, modelling studies are likely to be counterfactual. According to one optimistic projection, worldwide vaccine production will reach 16.47 billion doses in 2021 [19], but the actual annual production may be only approximately 10 billion doses. In addition, people with severe disease, low income and high-risk occupations (including frontline healthcare workers, security guards and transportation workers) should be prioritised for COVID-19 vaccination [20]. The conclusion that optimal vaccination for older people can minimise deaths and enhance the effectiveness of vaccines for younger people may change in an age-structured population [21]. Currently, country-level prioritisation for COVID-19 vaccines needs to be advocated.

### Scenario of equitable vaccine allocation strategy

Ideal vaccine allocation at the country level aims to accelerate the timetable for reaching global herd immunity. In accordance with the experimental design presented in section ‘Design of the experiments’, the modelling study reveals that, based on the homogeneous population assumption, if the 10 billion doses are allocated evenly, all countries could reach herd immunity by the end of 2021 (Fig. 3b, Table S2). Compared with the results shown in Figure 3a, the total number of required vaccine doses and cumulative cases in the ideal scenario are 17.88 billion (at least 10.95 billion doses are required if all countries/regions no longer administer vaccines after reaching herd immunity) and 238.82 million, respectively, by the end of 2021. This scenario indicates that vaccines are adequately allocated and that up to 22.19 million people are protected from the disease, which further implies that together with a more equitable allocation, the introduction of the remaining 18.72% of vaccines could contribute to a 9.29% reduction in infections. The evidence presented in Figure 4 shows that the average date of herd immunity decreases from 443 to 100 days, which suggests that the timetable is also substantially accelerated.

This scenario further suggests how COVID-19 vaccines should be prioritised at the country level. Because the countries/regions shown in deep red in Figure 3c have great potential to accelerate their achievement of herd immunity, providing sufficient vaccines to low-income countries – most of which are in Africa, South America, Eastern Europe, and South and Southeast Asia – could considerably reduce the time needed to achieve global herd immunity.

**Fig. 5.**
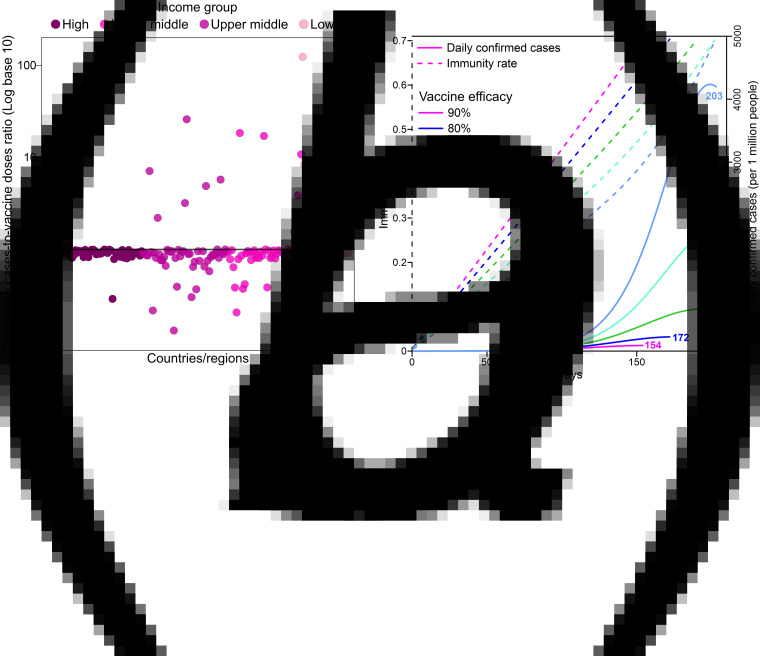
(a) Cumulative case-to-vaccine dose ratio grouped by income based on the current vaccinations administered. Ratios close to 1 indicate that the corresponding countries have small infected populations, whereas ratios far from 1 indicate that the countries have severe pandemic situations and large infected populations. (b) Days to herd immunity and daily confirmed cases based on different vaccine efficacy levels. This experiment assumes that 1% of the population is vaccinated each day. The time required to achieve herd immunity increases from 18 to 36, 48 and 49 days as the vaccine efficacy gradually decreases from 90% to 80%, 70%, 60% and 50%, respectively. The results may vary depending on the parameters in Eq. (1).

Figure 5a uses the cumulative case-to-vaccine dose ratio presented in section ‘Cumulative case-to-vaccine dose ratio’ to further demonstrate the intensity of the epidemic worldwide. Dozens of countries/regions have severe pandemic situations, which means that these countries are likely to reach herd immunity mainly through naturally acquired immunisation. Therefore, these countries are in great need of rapid vaccine assistance to control the spread of the disease.

### Scenario of SARS-CoV-2 variants

Even if global herd immunity is reached, the COVID-19 pandemic may not be contained soon. Virus mutations, for example, the SARS-CoV-2 Delta variant (B.1.617.2) [22], may negatively affect vaccine efficacy and naturally acquired immunity [23, 24]. If the effectiveness decreases from 90% to 80%, 70%, 60% and 50%, an additional 27.78%, 35.71%, 47.62% and 66.67% vaccine doses will be necessary, as determined using Eq. (2) presented in section ‘Model of the vaccine efficacy level’, and the epidemic dynamics in Eq. (1) indicate that 1, 13, 31 and 49 more days (Fig. 5b), respectively, will be needed to reach herd immunity. In this synthesis test, we assume that 1% of the population is vaccinated each day and that the initial infected population is 5 per 1 million people. The numerical values in this synthesis test may change according to the parameters in Eq. (1).

We now assess the perspective of the global COVID-19 pandemic caused by the Delta variant, which means that the vaccine effectiveness against the virus after full vaccination is approximately 70% (see the changes in the vaccine effectiveness levels in Eq. (1)). In the real world, an additional 52 countries/regions will likely take more than 3 years to reach herd immunity, and an additional 83 days will be required on average for countries to reach herd immunity in less than 3 years. Compared with the results obtained for the analysis of the pandemic due only to SARS-CoV-2, the number of total cumulative cases by the end of 2021 in the presence of virus mutations might increase by 113.37%, reaching 556.91 million. If a more equitable vaccine allocation is implemented, the total number of required vaccine doses equals 17.82 billion (at least 13.70 billion doses are needed if all countries/regions no longer administer vaccines after reaching herd immunity) by the end of 2021. This calculation implies that global herd immunity is theoretically impossible in 2021 because the number of required vaccine doses is markedly higher than the annual production. Compared with the pandemic caused only by SARS-CoV-2, although no additional vaccine doses are required, the number of cumulative cases increases by only 5.70%, reaching 252.43 million. This non-significant growth proves that equitable global vaccine allocation can play a key role in saving lives in the COVID-19 pandemic, even though the virus mutates and thereby reduces the effectiveness of the vaccines.

## Discussion

Under the current vaccination administration strategy, low-income countries may need more than 2 years to reach herd immunity, in contrast to the results obtained for high-income countries. If a more equitable vaccination strategy is executed, the world might reach herd immunity in the first half of 2022 as vaccine production expands. However, devastating virus variants may ruin the prospect of a rapid return to normal life. Decreases in the vaccine effectiveness lead to a higher probability of breakthrough COVID-19 infections; i.e. fully vaccinated people are not completely immune and become more susceptible [25]. The timetable of global herd immunity is therefore seriously delayed and fraught with uncertainty. A return to normal life was considered possible before the Delta variant arose. However, our study implies that, even in theory, human society cannot reach global herd immunity by the end of 2021. In fact, humans might find it difficult to achieve global herd immunity if no new vaccine with higher efficacy is produced or if a new SARS-CoV-2 variant with ultrafast transmissibility proliferates worldwide.

However, we further conclude that the introduction of a more equitable vaccine allocation strategy can contribute to non-significant growth in infections during all stages of the pandemic lifecycle. This model-informed study not only suggests the prospect of a rapid end to the COVID-19 pandemic but will also benefit decision-making regarding global vaccination administration, i.e. providing rapid vaccine assistance to low-income countries/regions can substantially accelerate the achievement of herd immunity and a return to normal life.

Although our scenario-based analysis suggests that the date of global herd immunity can be advanced, the ideal vaccination strategy included in the simulation is difficult to achieve in view of the current vaccine allocation strategy and vaccine hesitancy [26, 27]. Manageable disease occurs long after the achievement of herd immunity. High population flows could still lead to regional or even global outbreaks [28], which means that the rapid restoration of normal multilateral movements is impossible. Additionally, reaching global herd immunity will require deliberate policies. An equitable allocation of vaccines led by an authority (such as COVAX) could help promote the rapid achievement of global herd immunity. Moreover, although we suggest that a more equitable allocation could save many lives, vaccination is not the only way to contain the COVID-19 pandemic in the presence of virus variants and reduced vaccine effectiveness [27, 29, 30]. To save lives and accelerate global herd immunity, we recommend a more equitable vaccine allocation strategy, avoidance of overconsumption, prohibitions on vaccine hoarding and speculation, caution against the overrelaxation of control measures, and the provision of financial and medical assistance to low-income countries.

It is worth noting that this study has several limitations. (1) We provide a global model of the COVID-19 pandemic based on a series of homogeneous assumptions, including epidemic dynamics among the population without classification by age or occupational risk and the same vaccine effectiveness for different countries (even though effectiveness is a random value obtained over a specified interval). These homogeneous assumptions can be further improved by advanced technologies such as contact networks or agent-based models or by conducting a study with finer-grained settings. (2) The predictions for a couple of years also have significant uncertainty because the predictability of an outbreak usually decreases over an increasing time scale. (3) This study also does not consider booster shots and vaccine expiration dates, both of which may have impacts on vaccine effectiveness, but the exact real values remain unclear. We believe these two factors generally cannot qualitatively affect the final results of the COVID-19 pandemic in 2021 because only a minority of people will be offered booster shots or experience reduced effectiveness in 2021. (4) The end of the quarantine period, which may have significant impacts on the final results, is not considered in Eq. (1). Determining when to end quarantine is mainly dependent on the public health policy, which varies among countries and among the pandemic phases. To compensate for this deficit, we used an alternative strategy of estimating the quarantined population each day in the epidemic model. (5) Similarly, the exchange ratio between the quarantined and susceptible populations is also intrinsic in our model because both of those populations originate from the active population according to the time-dependent protection proportion. (6) The results may vary with the data acquisition time because the vaccinations administered are always changing.

## Conclusions

The ongoing vaccination rollout speeds worldwide vary among countries/regions and based on the existing socio-economic inequalities. Additionally, SARS-CoV-2 variants have arisen and spread globally, which leads to the waning of herd immunity and a delay in the return to normal life. We conducted a modelling study to reveal that a more equitable COVID-19 vaccine allocation could accelerate the achievement of global herd immunity and prevent more susceptible populations from being infected. Based on the actual annual vaccine production, the study further shows that global herd immunity is theoretically impossible by the end of 2021 even if a more equitable allocation strategy is implemented because the Delta variant reduces the vaccine effectiveness levels. Currently, returning to a normal pre-COVID-19 life is difficult, and herd immunity could easily fade as SARS-CoV-2 variants hint at a maximum risk. Comprehensive risk assessments and prevention plans are also critical for global herd immunity and sustained economic recovery. This study advances the scientific understanding of vaccine allocation, the fight against the spread of virus variants and the path to normal life.

## Data Availability

All data are available in the Supplementary materials.

## Supplementary material

For supplementary material accompanying this paper visit https://doi.org/10.1017/S0950268822000139. click here to view supplementary material
