# Supplementary material for: Return to normal pre-COVID-19 life is delayed by inequitable vaccine allocation and SARS-CoV-2 variants
Source: Epidemiol Infect. 2022 Jan 24;150:e46. doi: 10.1017/S0950268822000139 (PMC9058660; doi:10.1017/S0950268822000139)
Supplement: Supplementary file 1 [file S0950268822000139sup001.docx]

Supplementary Materials

Table A1.

**Timetable for 191 countries/regions to reach herd immunity** under the current phase of the pandemic and vaccinations administered (biweekly averaged data from 30 August 2021 to 12 September 2021) (the symbol “-” indicates countries that cannot reach herd immunity in 3 years or are in great need of rapid vaccine assistance to control their severe pandemic situations)

| **Continents** | **Countries/regions** | **Population** | **Date of herd immunity** | **Vaccine doses required** | **Total vaccinations per 100 people** |
| --- | --- | --- | --- | --- | --- |
| **South America** | Argentina | 45,605,823 | 2021/10/22 | 59,140,291 | 102.38 |
|  | Bolivia | 11,832,936 | 2022/11/3 | 17,848,828 | 52.76 |
|  | Brazil | 213,993,441 | 2021/11/2 | 279,547,211 | 98.26 |
|  | Chile | 19,212,362 | Reached | 29,709,026 | 157.19 |
|  | Colombia | 51,265,841 | 2022/5/6 | 67,622,906 | 72.24 |
|  | Ecuador | 17,888,474 | 2021/10/27 | 23,354,548 | 113.33 |
|  | Guyana | 790,329 | 2022/5/25 | 1,101,813 | 63.53 |
|  | Paraguay | 7,219,641 | 2022/7/31 | 10,002,656 | 59.16 |
|  | Peru | 33,359,415 | 2022/3/23 | 45,313,310 | 61.90 |
|  | Suriname | 591,798 | 2022/3/17 | 778,337 | 61.68 |
|  | Uruguay | 3,485,152 | 2021/9/13 | 5,810,728 | 169.83 |
|  | Venezuela | 28,704,947 | - | - | - |
| **Oceania** | Australia | 25,788,217 | 2021/11/22 | 38,970,543 | 87.15 |
|  | Fiji | 902,899 | 2021/11/21 | 1,265,522 | 95.93 |
|  | Marshall Islands | 59,618 | - | - | - |
|  | New Zealand | 4,860,642 | 2021/11/8 | 7,298,319 | 85.66 |
|  | Papua New Guinea | 9,119,005 | - | - | - |
|  | Samoa | 200,144 | 2022/3/15 | 305,096 | 68.93 |
|  | Solomon Islands | 703,995 | 2023/7/16 | 1,049,249 | 11.85 |
|  | Togo | 8,478,242 | - | - | - |
|  | Vanuatu | 314,464 | - | - | - |
| **North America** | Antigua and Barbuda | 98,728 | 2022/4/19 | 142,286 | 79.00 |
|  | Bahamas | 396,914 | 2022/8/12 | 516,488 | 40.72 |
|  | Barbados | 287,708 | 2022/3/22 | 392,893 | 75.08 |
|  | Belize | 404,915 | 2022/1/4 | 562,628 | 63.19 |
|  | Canada | 38,067,913 | 2021/10/2 | 55,587,083 | 142.74 |
|  | Costa Rica | 5,139,053 | 2021/11/15 | 6,781,764 | 89.13 |
|  | Cuba | 11,317,498 | 2021/9/16 | 15,267,741 | 139.83 |
|  | Dominica | 72,172 | 2022/4/29 | 56,986 | 60.13 |
|  | Dominican Republic | 10,953,714 | 2022/3/3 | 15,840,861 | 104.48 |
|  | El Salvador | 6,518,500 | 2021/11/9 | 9,889,729 | 105.74 |
|  | Grenada | 113,015 | 2021/11/7 | 57,364 | 40.22 |
|  | Guatemala | 18,249,868 | 2022/7/3 | 26,752,100 | 29.89 |
|  | Haiti | 11,541,683 | - | - | - |
|  | Honduras | 10,062,994 | 2022/8/6 | 15,046,362 | 44.98 |
|  | Jamaica | 2,973,462 | 2022/11/27 | 3,773,620 | 20.65 |
|  | Mexico | 130,262,220 | 2022/5/2 | 197,671,931 | 70.00 |
|  | Nicaragua | 6,702,379 | - | - | - |
|  | Panama | 4,381,583 | 2021/10/7 | 5,763,385 | 111.03 |
|  | Saint Kitts and Nevis | 53,546 | 2022/6/29 | 72,472 | 87.00 |
|  | Saint Lucia | 184,401 | - | - | - |
|  | Saint Vincent and the Grenadines | 111,269 | - | - | - |
|  | Trinidad and Tobago | 1,403,374 | 2022/3/20 | 2,051,765 | 69.94 |
|  | US | 332,915,074 | 2021/11/5 | 415,407,370 | 113.98 |
| **Europe** | Albania | 2,872,934 | 2022/4/28 | 3,897,583 | 55.33 |
|  | Andorra | 77,354 | Reached | 93,430 | 120.78 |
|  | Austria | 9,043,072 | 2022/2/6 | 12,254,176 | 117.46 |
|  | Belarus | 9,442,867 | - | - | - |
|  | Belgium | 11,632,334 | Reached | 16,327,333 | 140.58 |
|  | Bosnia and Herzegovina | 3,263,459 | 2022/9/20 | 4,445,489 | 32.49 |
|  | Bulgaria | 6,896,655 | - | - | - |
|  | Croatia | 4,081,657 | 2022/11/16 | 5,407,878 | 81.51 |
|  | Cyprus | 888,005 | 2021/9/16 | 1,119,295 | 125.83 |
|  | Czechia | 10,724,553 | 2021/12/21 | 13,000,835 | 108.45 |
|  | Denmark | 5,813,302 | Reached | 8,649,137 | 149.22 |
|  | Estonia | 1,325,188 | 2022/1/13 | 1,679,779 | 98.74 |
|  | Finland | 5,548,361 | 2021/10/26 | 8,282,429 | 129.50 |
|  | France | 67,564,251 | Reached | 90,255,124 | 134.47 |
|  | Germany | 83,900,471 | 2021/12/21 | 120,316,529 | 123.93 |
|  | Greece | 10,370,747 | 2021/12/31 | 14,326,310 | 112.78 |
|  | Holy See | 812 | - | - | - |
|  | Hungary | 9,634,162 | 2023/11/9 | 9,249,943 | 96.01 |
|  | Iceland | 343,360 | Reached | 530,720 | 155.93 |
|  | Ireland | 4,982,904 | Reached | 6,951,868 | 140.60 |
|  | Italy | 60,367,471 | 2021/9/27 | 83,230,280 | 133.77 |
|  | Kosovo | 1,932,774 | 2021/12/24 | 2,450,371 | 52.55 |
|  | Latvia | 1,866,934 | 2022/5/3 | 2,532,703 | 83.19 |
|  | Liechtenstein | 38,254 | 2023/6/14 | 50,270 | 113.18 |
|  | Lithuania | 2,689,862 | 2021/10/9 | 3,467,362 | 121.48 |
|  | Luxembourg | 634,814 | 2021/10/31 | 805,651 | 121.53 |
|  | Malta | 514,564 | Reached | 801,271 | 156.53 |
|  | Moldova | 4,024,025 | - | - | - |
|  | Monaco | 39,520 | 2021/10/31 | 53,521 | 124.65 |
|  | Montenegro | 628,051 | 2022/1/15 | 622,856 | 66.20 |
|  | Netherlands | 17,173,094 | 2021/9/29 | 22,249,609 | 128.70 |
|  | North Macedonia | 2,082,661 | 2022/2/27 | 2,767,660 | 65.21 |
|  | Norway | 5,465,629 | 2021/10/4 | 8,058,725 | 135.17 |
|  | Poland | 37,797,000 | 2022/11/15 | 53,154,785 | 97.20 |
|  | Portugal | 10,167,923 | Reached | 15,153,637 | 150.64 |
|  | Romania | 19,127,772 | - | - | - |
|  | Russia | 145,912,022 | 2022/7/30 | 206,927,997 | 58.33 |
|  | San Marino | 34,010 | Reached | 46,568 | 136.94 |
|  | Serbia | 6,804,596 | 2022/1/6 | 8,000,786 | 89.50 |
|  | Slovakia | 5,460,726 | 2023/9/1 | 7,636,310 | 84.28 |
|  | Slovenia | 2,078,723 | 2022/1/6 | 2,544,998 | 94.35 |
|  | Spain | 46,745,211 | Reached | 67,582,722 | 145.91 |
|  | Sweden | 10,160,159 | 2021/9/28 | 13,215,105 | 129.31 |
|  | Switzerland | 8,715,494 | 2021/12/18 | 11,579,814 | 112.84 |
|  | Ukraine | 43,466,822 | 2022/11/20 | 63,682,259 | 24.54 |
|  | United Kingdom | 68,207,114 | Reached | 91,937,802 | 135.31 |
| **Asia** | Bhutan | 39,835,428 | - | - | - |
|  | Afghanistan | 2,968,128 | - | - | - |
|  | Armenia | 10,223,344 | 2022/1/10 | 14,138,335 | 73.29 |
|  | Azerbaijan | 1,748,295 | Reached | 2,511,607 | 144.09 |
|  | Bahrain | 166,303,494 | 2022/10/24 | 265,643,457 | 20.27 |
|  | Bangladesh | 779,900 | 2022/5/19 | 1,191,628 | 134.00 |
|  | Brunei | 441,532 | 2021/11/20 | 665,318 | 84.14 |
|  | Burma | 54,806,014 | - | - | - |
|  | Cambodia | 16,946,446 | 2021/10/24 | 25,933,786 | 121.99 |
|  | China Hong Kong | 7,552,800 | 2021/12/5 | 11,802,251 | 107.45 |
|  | China Macao | 658,391 | 2022/5/6 | 1,031,681 | 92.31 |
|  | China Taiwan | 23,855,008 | 2022/4/24 | 34,102,599 | 51.57 |
|  | Georgia | 3,979,773 | 2022/2/23 | 4,489,963 | 36.01 |
|  | India | 1,393,409,033 | 2022/3/12 | 1,992,185,199 | 52.64 |
|  | Indonesia | 276,361,788 | 2022/5/15 | 408,908,884 | 41.17 |
|  | Iran | 85,028,760 | 2022/10/21 | 115,042,956 | 34.29 |
|  | Iraq | 41,179,351 | - | - | - |
|  | Israel | 8,789,776 | Reached | 14,283,028 | 163.74 |
|  | Japan | 126,050,796 | 2021/11/5 | 189,732,190 | 112.20 |
|  | Jordan | 10,269,022 | 2022/10/10 | 14,093,998 | 64.66 |
|  | Kazakhstan | 18,994,958 | 2022/3/27 | 26,594,530 | 68.00 |
|  | Korea South | 51,305,184 | 2021/10/26 | 78,493,609 | 101.03 |
|  | Kuwait | 4,328,553 | - | - | - |
|  | Kyrgyzstan | 6,628,347 | - | - | - |
|  | Laos | 7,379,358 | 2022/11/13 | 11,109,307 | 59.66 |
|  | Lebanon | 6,769,151 | 2023/1/24 | 8,881,263 | 38.42 |
|  | Malaysia | 32,776,195 | 2021/10/12 | 45,598,279 | 116.73 |
|  | Maldives | 543,620 | Reached | 693,398 | 129.38 |
|  | Mongolia | 3,329,282 | 2021/10/26 | 4,466,785 | 130.92 |
|  | Nepal | 29,674,920 | 2022/6/6 | 45,123,453 | 36.83 |
|  | Oman | 5,223,376 | 2021/11/23 | 7,412,574 | 77.27 |
|  | Pakistan | 225,199,929 | 2022/6/13 | 337,860,560 | 29.90 |
|  | Palestine | 5,222,756 | 2022/3/7 | 6,358,120 | 32.05 |
|  | Philippines | 111,046,910 | 2022/9/21 | 159,648,815 | 33.97 |
|  | Qatar | 2,930,524 | Reached | 4,547,618 | 156.24 |
|  | Saudi Arabia | 35,340,680 | 2021/11/29 | 54,046,360 | 111.35 |
|  | Singapore | 5,896,684 | 2021/9/18 | 9,026,207 | 152.23 |
|  | Sri Lanka | 21,497,306 | 2021/10/16 | 32,656,891 | 110.61 |
|  | Syria | 18,275,704 | - | - | - |
|  | Tajikistan | 9,749,625 | 2022/3/24 | 15,840,711 | 32.56 |
|  | Thailand | 69,950,844 | 2022/1/6 | 101,526,488 | 55.57 |
|  | The Mainland of China | 1,444,216,102 | 2021/9/30 | 2,239,025,143 | 148.36 |
|  | Timor-Leste | 1,343,875 | 2023/5/20 | 1,972,752 | 43.78 |
|  | Turkey | 85,042,736 | 2021/10/9 | 114,277,827 | 119.30 |
|  | United Arab Emirates | 9,991,083 | Reached | 18,496,497 | 188.78 |
|  | Uzbekistan | 33,935,765 | 2022/6/16 | 53,952,525 | 47.08 |
|  | Vietnam | 98,168,829 | 2022/4/7 | 132,060,772 | 27.61 |
|  | Yemen | 30,490,639 | - | - | - |
| **Africa** | Algeria | 44,616,626 | 2022/4/25 | 69,937,823 | 17.93 |
|  | Angola | 33,933,611 | - | - | - |
|  | Benin | 12,451,031 | - | - | - |
|  | Botswana | 2,397,240 | 2022/5/30 | 3,110,304 | 23.93 |
|  | Burkina Faso | 21,497,097 | - | - | - |
|  | Burundi | 12,255,429 | - | - | - |
|  | Cabo Verde | 561,901 | 2022/11/6 | 684,751 | 54.99 |
|  | Cameroon | 27,224,262 | - | - | - |
|  | Central African Republic | 4,919,987 | - | - | - |
|  | Chad | 16,914,985 | - | - | - |
|  | Comoros | 888,456 | - | - | - |
|  | Congo (Brazzaville) | 5,657,017 | - | - | - |
|  | Congo (Kinshasa) | 92,377,986 | - | - | - |
|  | Cote d'Ivoire | 27,053,629 | - | - | - |
|  | Djibouti | 1,002,197 | - | - | - |
|  | Egypt | 104,258,327 | - | - | - |
|  | Equatorial Guinea | 1,449,891 | - | - | - |
|  | Eritrea | 3,601,462 | - | - | - |
|  | Eswatini | 1,172,369 | - | - | - |
|  | Ethiopia | 117,876,226 | - | - | - |
|  | Gabon | 2,278,829 | - | - | - |
|  | Gambia | 2,486,937 | - | - | - |
|  | Ghana | 31,732,128 | - | - | - |
|  | Guinea-Bissau | 2,015,490 | - | - | - |
|  | Guinea | 13,497,237 | - | - | - |
|  | Kenya | 54,985,702 | - | - | - |
|  | Lesotho | 2,159,067 | - | - | - |
|  | Liberia | 5,180,208 | - | - | - |
|  | Libya | 6,958,538 | 2022/12/21 | 9,202,088 | 18.31 |
|  | Madagascar | 28,427,333 | - | - | - |
|  | Malawi | 19,647,681 | - | - | - |
|  | Mali | 20,855,724 | - | - | - |
|  | Mauritania | 4,775,110 | - | - | - |
|  | Mauritius | 1,273,428 | 2021/11/7 | 1,944,638 | 124.97 |
|  | Morocco | 37,344,787 | 2021/12/7 | 55,561,632 | 97.13 |
|  | Mozambique | 32,163,045 | - | - | - |
|  | Namibia | 2,587,344 | - | - | - |
|  | Niger | 25,130,810 | - | - | - |
|  | Nigeria | 211,400,704 | - | - | - |
|  | Rwanda | 13,276,517 | 2023/3/2 | 20,570,547 | 19.92 |
|  | Sao Tome and Principe | 223,364 | 2023/2/13 | 306,642 | 22.78 |
|  | Senegal | 17,196,308 | - | - | - |
|  | Seychelles | 98,910 | Reached | 145,772 | 147.22 |
|  | Sierra Leone | 8,141,343 | - | - | - |
|  | Somalia | 16,359,500 | - | - | - |
|  | South Africa | 60,041,996 | 2022/9/21 | 86,790,586 | 24.43 |
|  | South Sudan | 11,381,377 | - | - | - |
|  | Sudan | 44,909,351 | - | - | - |
|  | Tanzania | 61,498,438 | - | - | - |
|  | Tunisia | 11,935,764 | 2022/4/28 | 16,918,714 | 52.72 |
|  | Uganda | 47,123,533 | - | - | - |
|  | Zambia | 18,920,657 | - | - | - |
|  | Zimbabwe | 15,092,171 | 2022/12/26 | 22,846,434 | 31.05 |

Table A2.

**The total number of vaccine doses required and the number of cumulative cases to accelerate the timetable to reach herd immunity to ensure that all countries can reach herd immunity by the end of 2021**, under the current phase of the pandemic and vaccinations administered (biweekly averaged data from 30 August 2021 to 12 September 2021)

| **Continents** | **Countries/regions** | **Current vaccinations administered** | | **Equitable vaccine allocation** | |
| --- | --- | --- | --- | --- | --- |
|  |  | **Cumulative cases** | **Vaccine doses required** | **Cumulative cases** | **Vaccine doses required** |
| **South America** | Argentina | 5,356,696 | 84,467,681 | 5,280,604 | 122,762,786 |
|  | Bolivia | 513,938 | 9,238,464 | 502,157 | 28,076,976 |
|  | Brazil | 21,826,000 | 368,950,628 | 21,320,915 | 575,554,305 |
|  | Chile | 1,661,084 | 41,812,830 | 1,642,068 | 29,597,982 |
|  | Colombia | 5,030,056 | 50,865,266 | 4,973,117 | 125,834,785 |
|  | Ecuador | 515,210 | 28,050,965 | 509,306 | 53,713,275 |
|  | Guyana | 38,250 | 751,265 | 31,028 | 1,961,692 |
|  | Paraguay | 465,735 | 6,113,592 | 461,619 | 17,166,269 |
|  | Peru | 2,216,624 | 34,515,175 | 2,183,840 | 80,355,533 |
|  | Suriname | 59,379 | 604,807 | 41,803 | 1,437,037 |
|  | Uruguay | 391,471 | 9,170,116 | 386,061 | 5,779,908 |
|  | Venezuela | 408,677 | 9,342,817 | 388,047 | 38,651,723 |
| **Oceania** | Australia | 249,864 | 48,849,382 | 139,663 | 70,715,061 |
|  | Fiji | 60,393 | 1,516,933 | 53,057 | 2,478,010 |
|  | Marshall Islands | 4 | 0 | 4 | 81,472 |
|  | New Zealand | 5,251 | 10,402,541 | 4,741 | 13,349,654 |
|  | Papua New Guinea | 18,785 | 302,524 | 18,445 | 12,572,495 |
|  | Samoa | 3 | 236,392 | 3 | 521,204 |
|  | Solomon Islands | 20 | 241,535 | 20 | 975,143 |
|  | Togo | 41,070 | 1,566,693 | 33,572 | 11,644,002 |
|  | Vanuatu | 4 | 43,645 | 4 | 431,479 |
| **North America** | Antigua and Barbuda | 3,473 | 108,831 | 2,305 | 262,196 |
|  | Bahamas | 26,489 | 278,488 | 21,835 | 892,292 |
|  | Barbados | 13,673 | 316,267 | 7,869 | 757,841 |
|  | Belize | 23,277 | 551,636 | 19,851 | 999,071 |
|  | Canada | 1,686,258 | 63,399,025 | 1,559,983 | 124,486,130 |
|  | Costa Rica | 594,582 | 8,580,982 | 529,379 | 13,283,889 |
|  | Cuba | 899,715 | 31,053,223 | 838,209 | 35,190,019 |
|  | Dominica | 12,944 | 49,633 | 3,935 | 178,426 |
|  | Dominican Republic | 370,523 | 14,207,214 | 357,402 | 31,805,463 |
|  | El Salvador | 101,140 | 12,816,623 | 98,637 | 19,041,820 |
|  | Grenada | 53,116 | 68,843 | 17,879 | 261,020 |
|  | Guatemala | 728,602 | 13,099,471 | 649,556 | 23,878,771 |
|  | Haiti | 23,372 | 155,581 | 22,379 | 15,786,434 |
|  | Honduras | 422,878 | 8,054,230 | 382,778 | 23,309,521 |
|  | Jamaica | 144,018 | 1,374,035 | 127,735 | 3,864,019 |
|  | Mexico | 4,344,327 | 139,868,357 | 3,792,611 | 332,922,095 |
|  | Nicaragua | 17,967 | 1,373,268 | 14,471 | 9,144,080 |
|  | Panama | 469,160 | 9,339,061 | 465,819 | 12,292,685 |
|  | Saint Kitts and Nevis | 3,473 | 56,272 | 1,597 | 147,432 |
|  | Saint Lucia | 26,306 | 65,631 | 13,704 | 403,077 |
|  | Saint Vincent and the Grenadines | 2,887 | 38,494 | 2,731 | 147,950 |
|  | Trinidad and Tobago | 59,364 | 1,585,778 | 52,044 | 3,561,140 |
|  | US | 48,116,004 | 457,841,314 | 42,127,321 | 940,307,892 |
| **Europe** | Albania | 224,978 | 2,677,033 | 182,455 | 6,799,098 |
|  | Andorra | 15,430 | 93,430 | 15,083 | 93,430 |
|  | Austria | 808,359 | 11,825,412 | 726,582 | 26,682,498 |
|  | Belarus | 649,464 | 4,042,869 | 600,090 | 12,034,256 |
|  | Belgium | 1,328,880 | 17,952,492 | 1,200,665 | 16,312,423 |
|  | Bosnia and Herzegovina | 260,642 | 1,965,418 | 249,105 | 4,074,964 |
|  | Bulgaria | 561,668 | 3,277,958 | 524,096 | 8,788,149 |
|  | Croatia | 425,390 | 3,840,975 | 393,796 | 10,461,188 |
|  | Cyprus | 130,474 | 1,364,450 | 120,448 | 2,603,607 |
|  | Czechia | 1,694,217 | 13,144,135 | 1,684,805 | 29,051,788 |
|  | Denmark | 394,059 | 9,809,076 | 350,533 | 8,638,495 |
|  | Estonia | 167,973 | 1,638,762 | 151,630 | 3,574,586 |
|  | Finland | 164,554 | 10,159,936 | 142,743 | 17,503,946 |
|  | France | 7,078,667 | 118,755,541 | 6,690,232 | 89,993,652 |
|  | Germany | 4,692,609 | 122,037,571 | 4,182,607 | 257,508,280 |
|  | Greece | 780,551 | 14,326,310 | 649,635 | 30,433,288 |
|  | Holy See | 27 | 0 | 27 | 1,047 |
|  | Hungary | 829,852 | 9,249,943 | 818,092 | 26,302,589 |
|  | Iceland | 14,360 | 599,725 | 11,158 | 530,087 |
|  | Ireland | 411,662 | 8,540,769 | 371,025 | 15,876,049 |
|  | Italy | 4,891,891 | 106,974,831 | 4,688,989 | 187,337,489 |
|  | Kosovo | 228,126 | 2,554,198 | 190,712 | 4,362,302 |
|  | Latvia | 167,310 | 2,003,935 | 152,831 | 4,860,198 |
|  | Liechtenstein | 4,028 | 44,365 | 3,526 | 110,634 |
|  | Lithuania | 344,056 | 4,231,354 | 316,259 | 7,832,088 |
|  | Luxembourg | 81,811 | 851,884 | 77,517 | 1,848,198 |
|  | Malta | 40,172 | 877,493 | 36,587 | 800,572 |
|  | Moldova | 309,182 | 1,761,402 | 299,142 | 5,064,621 |
|  | Monaco | 3,678 | 59,534 | 3,349 | 118,810 |
|  | Montenegro | 157,201 | 596,695 | 133,726 | 1,388,929 |
|  | Netherlands | 2,152,840 | 23,239,175 | 2,022,099 | 51,483,331 |
|  | North Macedonia | 224,186 | 2,254,178 | 197,761 | 4,957,020 |
|  | Norway | 244,640 | 11,356,594 | 195,352 | 17,472,165 |
|  | Poland | 2,924,073 | 40,789,388 | 2,900,462 | 104,010,221 |
|  | Portugal | 1,110,670 | 20,473,662 | 1,050,111 | 15,104,829 |
|  | Romania | 1,211,854 | 10,702,231 | 1,155,338 | 44,659,145 |
|  | Russia | 8,168,092 | 125,413,709 | 7,610,854 | 351,583,917 |
|  | San Marino | 5,385 | 47,246 | 5,349 | 46,562 |
|  | Serbia | 1,093,988 | 7,898,480 | 893,935 | 17,653,259 |
|  | Slovakia | 411,034 | 5,052,280 | 401,519 | 14,376,377 |
|  | Slovenia | 305,255 | 2,512,754 | 282,275 | 5,415,745 |
|  | Spain | 5,118,119 | 89,077,546 | 4,901,111 | 67,385,522 |
|  | Sweden | 1,138,875 | 16,829,748 | 1,133,596 | 30,051,842 |
|  | Switzerland | 817,677 | 11,825,584 | 792,523 | 25,055,193 |
|  | Ukraine | 2,560,384 | 23,548,934 | 2,523,445 | 55,095,537 |
|  | United Kingdom | 10,111,682 | 103,916,092 | 7,465,362 | 91,827,909 |
| **Asia** | Bhutan | 157,213 | 8,095,385 | 156,511 | 53,565,993 |
|  | Afghanistan | 287,292 | 524,794 | 273,270 | 3,570,170 |
|  | Armenia | 718,919 | 13,563,172 | 560,911 | 26,138,777 |
|  | Azerbaijan | 278,908 | 2,801,967 | 273,293 | 2,508,943 |
|  | Bahrain | 1,736,304 | 92,282,160 | 1,670,049 | 225,814,630 |
|  | Bangladesh | 2,608 | 1,109,817 | 2,599 | 2,547,862 |
|  | Brunei | 11,150 | 850,644 | 6,991 | 1,200,144 |
|  | Burma | 778,241 | 6,253,687 | 631,211 | 73,329,164 |
|  | Cambodia | 121,172 | 35,444,698 | 108,945 | 52,636,260 |
|  | China Hong Kong | 12,227 | 13,013,925 | 12,174 | 22,466,244 |
|  | China Macao | 64 | 805,340 | 63 | 1,880,508 |
|  | China Taiwan | 16,459 | 22,577,280 | 16,223 | 57,545,016 |
|  | Georgia | 761,750 | 3,452,801 | 656,140 | 7,936,423 |
|  | India | 35,849,126 | 1,480,776,657 | 34,488,677 | 3,318,392,037 |
|  | Indonesia | 4,677,386 | 242,373,038 | 4,558,437 | 378,898,339 |
|  | Iran | 7,585,143 | 52,538,535 | 7,000,552 | 108,996,012 |
|  | Iraq | 2,428,512 | 8,749,455 | 2,256,792 | 52,162,972 |
|  | Israel | 1,127,408 | 21,356,746 | 1,113,207 | 14,218,131 |
|  | Japan | 2,428,400 | 245,780,798 | 1,954,364 | 376,299,945 |
|  | Jordan | 872,691 | 8,675,559 | 830,431 | 24,869,671 |
|  | Kazakhstan | 1,270,599 | 20,410,983 | 1,050,481 | 47,493,434 |
|  | Korea South | 331,303 | 124,442,121 | 310,187 | 146,538,156 |
|  | Kuwait | 419,067 | 2,375,455 | 413,433 | 9,950,368 |
|  | Kyrgyzstan | 189,987 | 2,249,809 | 185,105 | 8,731,033 |
|  | Laos | 37,308 | 6,115,742 | 22,602 | 18,618,105 |
|  | Lebanon | 682,199 | 3,934,322 | 661,356 | 8,695,405 |
|  | Malaysia | 2,916,856 | 69,206,187 | 2,443,402 | 96,476,600 |
|  | Maldives | 92,388 | 836,515 | 82,364 | 692,085 |
|  | Mongolia | 423,881 | 4,643,378 | 261,710 | 10,320,836 |
|  | Nepal | 869,570 | 24,785,617 | 847,873 | 39,438,868 |
|  | Oman | 306,166 | 9,194,470 | 304,852 | 13,549,388 |
|  | Pakistan | 1,547,019 | 173,536,543 | 1,454,378 | 309,729,883 |
|  | Palestine | 550,585 | 4,576,700 | 495,410 | 6,471,147 |
|  | Philippines | 4,101,522 | 72,364,343 | 3,547,750 | 148,554,551 |
|  | Qatar | 241,626 | 5,834,371 | 234,001 | 4,535,813 |
|  | Saudi Arabia | 556,555 | 60,520,653 | 548,706 | 105,536,062 |
|  | Singapore | 85,919 | 9,884,371 | 73,926 | 20,243,167 |
|  | Sri Lanka | 622,831 | 56,681,929 | 573,702 | 62,559,084 |
|  | Syria | 38,721 | 519,021 | 38,101 | 24,732,439 |
|  | Tajikistan | 18,637 | 10,393,599 | 18,383 | 13,555,567 |
|  | Thailand | 2,741,685 | 98,132,681 | 1,982,408 | 168,621,910 |
|  | The Mainland of China | 96,552 | 2,882,729,429 | 95,684 | 4,905,050,299 |
|  | Timor-Leste | 35,913 | 835,925 | 28,655 | 1,914,277 |
|  | Turkey | 7,552,809 | 164,373,651 | 7,054,205 | 249,748,611 |
|  | United Arab Emirates | 781,856 | 24,726,112 | 726,064 | 18,439,345 |
|  | Uzbekistan | 213,360 | 30,684,415 | 187,804 | 80,655,785 |
|  | Vietnam | 3,243,304 | 80,803,007 | 2,412,062 | 130,055,650 |
|  | Yemen | 12,826 | 311,483 | 10,992 | 41,970,837 |
| **Africa** | Algeria | 223,580 | 38,280,714 | 218,775 | 61,301,577 |
|  | Angola | 68,462 | 4,009,416 | 61,216 | 46,136,249 |
|  | Benin | 138,940 | 582,445 | 50,569 | 16,719,732 |
|  | Botswana | 204,153 | 1,620,515 | 192,435 | 2,912,061 |
|  | Burkina Faso | 14,869 | 188,384 | 14,565 | 29,469,222 |
|  | Burundi | 19,436 | 0 | 15,707 | 16,732,172 |
|  | Cabo Verde | 43,766 | 407,412 | 39,848 | 1,325,563 |
|  | Cameroon | 91,582 | 507,066 | 88,578 | 36,622,412 |
|  | Central African Republic | 11,499 | 137,157 | 11,421 | 6,653,816 |
|  | Chad | 5,083 | 261,121 | 5,055 | 23,178,579 |
|  | Comoros | 4,327 | 277,419 | 4,237 | 1,220,742 |
|  | Congo (Brazzaville) | 13,811 | 441,836 | 13,761 | 7,814,859 |
|  | Congo (Kinshasa) | 60,038 | 252,586 | 58,100 | 126,284,832 |
|  | Cote d'Ivoire | 76,066 | 2,979,765 | 69,020 | 36,539,013 |
|  | Djibouti | 12,284 | 111,024 | 12,123 | 1,347,664 |
|  | Egypt | 311,581 | 26,359,787 | 309,974 | 142,111,221 |
|  | Equatorial Guinea | 19,594 | 526,365 | 15,713 | 1,992,418 |
|  | Eritrea | 6,818 | 0 | 6,739 | 4,912,909 |
|  | Eswatini | 53,680 | 422,656 | 51,950 | 1,524,539 |
|  | Ethiopia | 459,126 | 5,076,443 | 398,708 | 159,320,544 |
|  | Gabon | 30,559 | 269,554 | 29,174 | 3,074,875 |
|  | Gambia | 10,696 | 261,748 | 10,335 | 3,420,170 |
|  | Ghana | 155,201 | 1,271,393 | 138,574 | 43,417,997 |
|  | Guinea-Bissau | 7,413 | 31,090 | 6,798 | 2,710,511 |
|  | Guinea | 34,695 | 2,887,403 | 32,996 | 18,344,804 |
|  | Kenya | 298,592 | 5,145,263 | 276,024 | 74,038,379 |
|  | Lesotho | 14,617 | 201,795 | 14,554 | 2,918,380 |
|  | Liberia | 6,949 | 113,680 | 6,408 | 7,012,988 |
|  | Libya | 431,871 | 3,039,896 | 400,056 | 8,656,067 |
|  | Madagascar | 43,670 | 959,512 | 43,334 | 39,075,173 |
|  | Malawi | 65,709 | 1,381,974 | 63,987 | 27,027,000 |
|  | Mali | 15,695 | 718,630 | 15,457 | 28,826,542 |
|  | Mauritania | 45,106 | 678,463 | 41,295 | 6,428,790 |
|  | Mauritius | 22,831 | 2,310,743 | 14,488 | 4,003,009 |
|  | Morocco | 1,130,977 | 61,154,068 | 984,066 | 106,001,983 |
|  | Mozambique | 171,215 | 2,297,842 | 162,790 | 43,916,910 |
|  | Namibia | 133,889 | 705,231 | 131,775 | 3,282,946 |
|  | Niger | 6,389 | 491,738 | 6,254 | 33,991,630 |
|  | Nigeria | 250,027 | 12,989,650 | 229,969 | 286,782,509 |
|  | Rwanda | 126,481 | 6,264,067 | 116,048 | 17,953,395 |
|  | Sao Tome and Principe | 3,856 | 105,092 | 3,508 | 300,983 |
|  | Senegal | 78,233 | 1,980,231 | 77,346 | 23,421,554 |
|  | Seychelles | 20,230 | 162,356 | 20,218 | 145,620 |
|  | Sierra Leone | 6,540 | 193,557 | 6,468 | 11,040,179 |
|  | Somalia | 25,293 | 525,943 | 22,283 | 22,098,757 |
|  | South Africa | 3,391,691 | 35,024,165 | 3,247,615 | 76,387,965 |
|  | South Sudan | 12,602 | 82,462 | 12,192 | 15,598,640 |
|  | Sudan | 40,329 | 1,129,054 | 39,324 | 60,957,688 |
|  | Tanzania | 1,390 | 304,603 | 1,382 | 84,350,600 |
|  | Tunisia | 805,761 | 11,384,573 | 736,244 | 27,993,062 |
|  | Uganda | 130,767 | 3,803,977 | 127,271 | 64,146,928 |
|  | Zambia | 218,696 | 1,210,144 | 215,124 | 25,544,012 |
|  | Zimbabwe | 137,590 | 8,741,454 | 134,062 | 20,894,606 |
